# Structural Covariance of Gray Matter Volume in HIV Vertically Infected Adolescents

**DOI:** 10.1038/s41598-018-19290-5

**Published:** 2018-01-19

**Authors:** Jielan Li, Lei Gao, Zhi Wen, Jing Zhang, Panying Wang, Ning Tu, Hao Lei, Fuchun Lin, Xi’en Gui, Guangyao Wu

**Affiliations:** 1 Department of Magnetic Resonance Imaging, Zhongnan Hospital of Wuhan University, Wuhan, China; 2 National Center for Magnetic Resonance in Wuhan, State Key Laboratory of Magnetic Resonance and Atomic and Molecular Physics, Wuhan Institute of Physics and Mathematics, Chinese Academy of Sciences, Wuhan, China; 3 Department of Infectious Diseases, Zhongnan Hospital of Wuhan University, Wuhan, China

## Abstract

Human immunodeficiency virus (HIV) infection significantly affect neurodevelopmental and behavioral outcomes. We investigated whether alterations of gray matter organization and structural covariance networks with vertical HIV infection adolescents exist, by using the GAT toolbox. MRI data were analysed from 25 HIV vertically infected adolescents and 33 HIV-exposed-uninfected control participants. The gray matter volume (GMV) was calculated, and structural brain networks were reconstructed from gray matter co-variance. Gray matter losses were pronounced in anterior cingulate cortex (ACC), right pallidum, right occipital lobe, inferior parietal lobe, and bilateral cerebellum crus. The global brain network measures were not significantly different between the groups; however, the nodal alterations were most pronounced in frontal, temporal, basal ganglia, cerebellum, and temporal lobes. Brain hubs in the HIV-infected subjects increased in number and tended to shift to sensorimotor and temporal areas. In the HIV-infected subjects, decreased GMVs in ACC and bilateral cerebellum were related to lower Mini-Mental State Examination scores; the CD4 counts were positively related to the GMVs in ACC and sensorimotor areas. These findings suggest that focally reduced gray matter, disrupted nodal profiles of structural wirings, and a shift in hub distribution may represent neuroanatomical biomarkers of HIV infection on the developing brain.

## Introduction

The mortality of perinatal infection with human immunodeficiency virus (HIV) has been transformed from a near-uniformly fatal condition to a chronic or manageable disease to date^1,2^. Consequently, there is an increasing population of vertically infected survivors. Highly active antiretroviral therapy (HAART) is imperative for long-term survival following vertical infection. However, HIV has been associated with negative long-term effects on neurocognitive functions, including subtle effects on executive functioning, visual-spatial memory processing, and motor functioning^3,4^. The potential mechanisms include a direct immuno-virologic impact, which leads to the atrophy of gray matter and the demyelination of white matter fibers, as well as secondary microvascular injury^5,6^.

Vertically infected adolescents carry HIV from birth. Thus, these children are chronically exposed to the effects of HIV during a period of rapid cognitive development and brain maturation. Jahanshad N *et al*.^7^ found that in HIV-exposure but uninfected children compared to HIV-unexposed and uninfected children, no differences in neuroanatomical or brain integrity measures were detectable. Advanced neuroimaging may enhance our understanding of the neuroanatomical involvement of HIV infection. In a pilot brain morphometric study, Aylward *et al*.^8^ investigated the volumetric differences in the brain between seropositive HIV and seronegative adults. The authors suggested that HIV infection causes generalized brain atrophy; however, the clinical features of HIV dementia develop with selective basal ganglia atrophy. Other recent research has demonstrated profound frontal and temporal gray matter atrophy in HIV-infected adults^9–12^. However, in the existing three reports of HIV-infected adolescents, the authors have reported inconsistent findings. Sarma *et al*.^13^ reported a volume increase in the frontal and temporal gray matter in vertically infected youth, whereas two additional studies^14,15^ identified cortical atrophy in the left supplementary motor area, frontal cortex, and anterior cingulate cortex (ACC).

Recently, increased attention has been directed at investigating the structural covariance of the connected gray matter neuroanatomy. The biological significance of these association matrixes remains vague; however, it has been demonstrated that the networks of gray matter covariance reflect the patterns of coordinated structural maturation^16,17^ and disease propagation effects^18^. Based on the association between the synchronized developmental changes in distributed cortical regions and inter-regional anatomical correlations patterns, it is hypothesized that vertically infected adolescents would exhibit differences in anatomical gray matter covariance particularly since the presence of HIV infection occurs during a critical period for the brain maturation and development (i.e., from the moment of birth or prenatally).

Preliminary findings have suggested that although HAART may effectively reduce the incidence of HIV encephalopathy and mortality in perinatal HIV-infected adolescents and may improve the quality of life, many children may exhibit continuous brain injury following early effective treatment^19^. Cognitive function and brain development are also affected in these children. We speculated that the structural brain network of perinatal HIV-infected adolescents would differ from that of HIV- adolescents. However, it has not been determined whether the organization differences of these gray-matter covariance networks are characteristic of vertically infected adolescents.

More recently, complex network analysis based on graph theory had been used to characterize various organizational features of brain function and structural networks^20^. These methods use characteristics of network dynamics to describe the brain as a complex network consisting of regions (i.e., nodes) and the connections between regions (i.e., edges)^21^. In this study, we investigated the organization differences in regional gray matter volume (GMV) covariance networks in HIV vertically infected adolescents. Specifically, we investigated whether HIV vertically infected adolescents exhibited differences in large-scale structural brain topology.

## Materials and Methods

### Participants

We recruited 25 HIV-positive adolescents (HIV+) (mean age ± SD, 15.0 ± 1.7 years; range, 12–17 years). The presence of HIV was confirmed by enzyme-linked immunosorbent assay (ELISA) and Western blot analysis. Also recruited 33 age- and gender-matched HIV-exposed but uninfected subjects (HIV−) (mean age ± SD, 14.8 ± 1.6 years; range, 12–18 years). All HIV-positive adolescents were infected by mother-to-child transmission during pregnancy, childbirth or through breastfeeding, and they were infected with the same clade/strain of the virus. HIV-negative subjects’ fathers, mothers, or parents also suffered from HIV infections. The socioeconomic status, cultural background and ethnic background of the two communities were similar. Detailed population information and clinical measures are listed in Table 1. All subjects were enrolled from the Center of AIDS Prevention and Cure of Zhongnan Hospital, Wuhan University. The inclusion criteria for HIV-infected subjects included HIV acquisition during fetal or neonatal period, currently treated with HAART, and right-handed. For the control subjects, the inclusion criteria included confirmation of HIV negative status by ELISA and right-handedness.

**Table 1:** Demographic information for subjects in each group.

	HIV(+)	HIV(−)	p value
Age (years)	15.0 ± 1.7	14.8 ± 1.6	0.7
Gender (male/female)	14/10	16/17	0.46
Education level (years)	8.0 ± 1.6	8.1 ± 1.9	0.84
Current CD4 (cells/mm^3^)	597.5 ± 257.7	NA	NA
Age at first HIV treatment	8.5 ± 3.3	NA	NA
HIV treatment duration (months)	75.9 ± 34.9	NA	NA
% treated at less than two year	16.7(n = 4)	NA	NA
Plasma viral load (copies/mL)	686.0 ± 3222.2	NA	NA
MMSE total score	25.9 ± 2.3	27.4 ± 2.0	0.006
Ethnicity	Han Chinese	Han Chinese	NA
Parental status	Farmer	Farmer	NA

Note: NA indicates not applicable or available; MMSE: Mini-Mental State Examination.

Exclusion criteria for all subjects included those younger than 12 years of age or over 18 years of age, with acute medical illnesses, current or past medical or neurological diseases, psychiatric illnesses, mental retardation, current alcohol or drug abuse, HIV encephalopathy and opportunistic infections, magnetic resonance imaging (MRI) contraindications, claustrophobia, metabolic disorders or other brain diseases (not AIDS-related). We used the exclusion criteria, which included HIV encephalopathy to rule out space-occupying masses, other lesions or cortical atrophy in the brain of HIV-infected adolescents, so that we got the difference in anatomical gray matter covariance between the two groups. For the control subjects, the exclusion criteria also included serious educational difficulties and a chronic medication other than asthma medication. Most HIV infection participants participated in laboratory evaluations, such as plasma CD4 T-cell counts.

The study was approved by the Medical Ethics Committee of Zhongnan Hospital of Wuhan University, and a written and informed consent was made from all participants or their guardians following a complete description of the measurements. These methods were carried out in accordance with the approved guidelines and regulations.

### Assessments

The Mini-Mental State Examination (MMSE) were used to assess the cognitive abilities of the subjects.

### MRI acquisition

High-resolution T1-weighted structural MRI scans were acquired on the 3.0 T scanner (Siemens, Tim-Trio; Erlangen, Germany), which was stationed at the Department of Radiology, the Zhongnan Hospital of Wuhan University, using a multi-echo MPRAGE pulse sequence (repetition time = 1900 ms, echo time = 2.1 ms, inversion time = 900 ms, flip angle = 9°, slice thickness = 1.00 mm, and matrix size = 320 × 320) that yielded 160 axial slices with an in-plane resolution of 1.0 × 1.0 mm. We visually inspected the cerebral microbleeds foci measured by susceptibility-weighted imaging (SWI), and white matter hyperintensity by T2- fluid-attenuated inversion recovery (FLAIR) images through all the subjects. We also excluded any subject that exhibited obvious gray and white matter lesion imaged by SWI and FLAIR.

### Voxel-based morphometry (VBM) analysis

VBM analyses were performed using Statistical Parametric Mapping software (SPM12; Wellcome Department of Cognitive Neurology, London, UK; http://www.fil.ion.ucl.ac.uk/spm) and CAT12 (CAT, http://dbm.neuro.uni-jena.de/vbm/) based on MATLAB (MathWorks, Natick, MA, USA). All T1 images were manually checked by an operator blind to subject identity. The processing steps were as follows: (1) the raw T1 images were manually reoriented to the center point of the AC-PC plane; (2) the reoriented images were segmented into white matter, gray matter and cerebrospinal fluid using the standard unified segmented model in CAT12; (3) the gray matter images were nonlinearly normalized into standard Montreal Neurological Institute space using a pediatric template for 12- to 18-year-old children from the Imaging Research Center at Cincinnati Children’s Hospital Medical Center (CCHMC); (4) these normalized sections were then adjusted to ensure that the relative volumes of gray matter were retained after the spatial normalization procedure; (5) the modulated images were re-sampled to 1.5 × 1.5 × 1.5 mm^3^ and smoothed with an 8 mm full-width at half-maximum (FWHM) Gaussian kernel; (6) to control for deviations, we included an additional quality check based on heterogeneity measurements of the sample as implemented in CAT; using the covariance of voxel-based data to identify the outliers who were two or more standard deviations outside of the GMV sample distributions, and one patient and one control were excluded based on this criterion; (7) finally, exclude voxels with a gray matter value <0.15 to eliminate the potential edge effects between the gray matter and white matter.

### Network construction

All graph analysis in the current study was performed using the GAT toolbox^22^. Brain parcellation was performed following the automated anatomical labeling (AAL) algorithm with 90 cortical and subcortical regions of interest (ROIs). The regional GMVs of 90 ROIs were extracted from the individual modulated normalized gray matter images in each group, where the age, gender and total brain size of the subject were included as covariates of nuisance.

A 90 × 90 association matrix R was constructed for each group using the pair-wise regional correlations calculated as the partial correlation coefficients (r_*ij*_) between the average GMV values for a pair of ROIs, “*i*” and “*j”*. The matrix R was subsequently transformed into a binary matrix A by thresholding the r_*ij*_ into values of 0 or 1. These thresholds were defined as the ranges of the network densities (*D*_*min*_*-D*_*max*_), where D_*min*_ represents the minimum density above which the networks were not fragmented^22^. In the current study, we initially calculated the network metrics across a density range of 0.1–0.45 (interval of density, 0.01) because structural networks with >50% connectivity are considered less biologically significant. Ultimately, the density region of interest for the global property comparisons was set to 0.13–0.45; above 0.13, the networks of the HIV+ and HIV− groups were fully connected (i.e., each node of the network had at least one connection with another node). For the comparisons of regional property, we chose the range of 0.13–0.45, in which the two networks groups were completely connected and showed differences between groups in small-worldness.

### Global network properties

We initially evaluated the differences within-group and between-group in global network measures, such as small-worldness, clustering, and path length. The small-worldness index was defined as [C/C_rand_]/[L/L_rand_], where C indicates the clustering coefficient, L represents the characteristic path length, and C_rand_ and L_rand_ are the average clustering coefficient and characteristic path length of 20 random networks, respectively. The small-world characteristics were calculated using the area under the curve (AUC) at a minimum connection density (0.13) and across a range of densities (0.13–0.45). When the minimum density of the small-worldness index was >1, the graph was considered small-world^22,23^.

### Regional network properties and hub identification

We subsequently quantified the within-group and between-group differences in the regional network properties, such as the normalized betweenness centrality bi, normalized degree ki, and the hubs:

1 $${b}_{i}={\sum }_{m\ne i\ne n\in G}\frac{{\sigma }_{mn}(i)}{{\sigma }_{mn}}$$

2 $${k}_{i}^{B}={\sum }_{j\in G}\,{a}_{ij}\,{\rm{or}}$$

3 $${k}_{i}^{W}={\sum }_{j\in G}\,{w}_{ij}$$

where *B*_*i*_ represents the total number of the shortest paths that pass through node i, *K*_*i*_ represents the number of edges between node i and other nodes, and *B* and *K* represent the average betweenness and degree of the entire network respectively. In addition, *b*_*i*_ and *k*_*i*_ were used to identify the hubs of the network; nodes with regional values of at least greater than the mean value one SD were identified as hubs.

### Statistical analysis

Statistical analysis for the patient demographics was conducted using IBM SPSS version 20 (IBM SPSS Inc., Chicago, IL, USA) and included Chi-square and independent sample t-tests for the participant characteristics. The significance threshold was set to p < 0.05. The whole brain GMV differences between the two groups were tested in CAT and SPM12 using two-sample t-tests, and the whole brain volume, age and gender were used as covariates of without interest. The significance threshold was set to p < 0.05 with a cluster level Family Wise Error (FWE) correction and threshold-free cluster enhancement (TFCE) multiple comparison-corrected.

## Results

### Demographic and clinical data

Table 1 shows the demographic and clinical data for all subjects. There were no significant differences in age or duration of education between the HIV-infected subjects and the HIV-exposed-uninfected group; however, the MMSE scores were significantly lower in the HIV-infected subjects (p = 0.006). The CD4 counts ranged from 144–1025 cells/mm^3^ (average: 597.5 cells/mm^3^). 18 HIV+ adolescents were with undetectable plasma viral loads.

### Differences in GMV and global GMV

**Figure 1:**
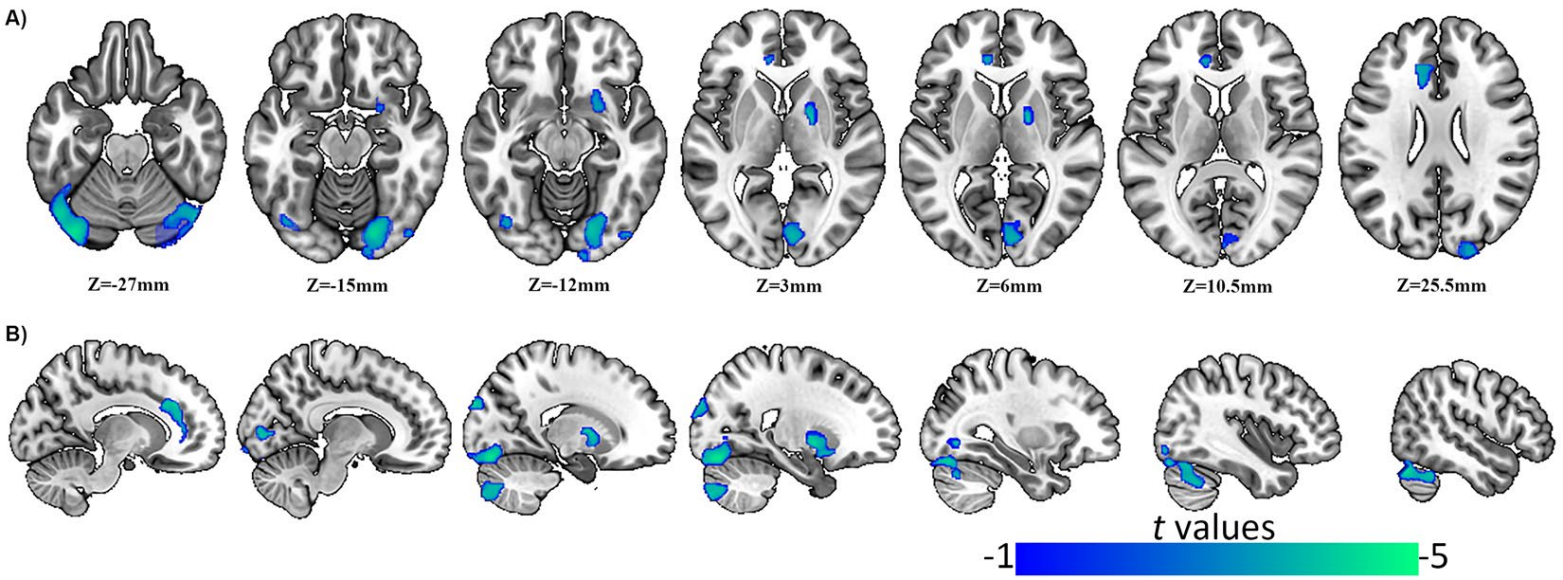
Differences in GMV. The picture on the left side corresponds to the left hemisphere. (**A**) GMV changes occur in the region with MNI co-ordinates in the z direction between z = −27 to z = 25.5. (**B**) Sagittal bitmaps indicate the relative reduced GMV regions.

GMV changes were identified in the HIV+ adolescents compared with the HIV− subjects and are shown in Fig. 1 and Table 2. HIV-positive adolescents showed significantly reduced GMVs (blue) in both bilateral cerebellum crus, right cerebellum, right pallidum, right calcarine, left anterior cingulate cortex, and right superior occipital lobe compared with control subjects. In the analysis, HIV-positive adolescents did not show a significantly increased GMV.

**Table 2:** Regions of GMV changes in HIV positive adolescents compared with age- and gender-matched controls.

Brain regions	MNI coordinate			Cluster size	BA	T value
	x	y	z			
Right cerebellum crus	19.5	−78	−42	508	—	−3.308
Left cerebellum crus	−36	−81	−22.5	2459	—	−4.385
Right cerebellum	19.5	−82.5	−15	2842	—	−4.051
Right pallidum	19.5	−4.5	3	548	—	−3.831
Right calcarine	7.5	−85.5	3	349	18/17	−3.474
Left ACC	−12	31.5	28.5	534	32/9	−3.509
Right superior occipital lobe	18	−96	30	275	19/18	−3.616

BA: Brodmann’s area; ACC: anterior cingulate cortex. P < 0.05, with a cluster level Family Wise Error (FWE) correction and threshold-free cluster enhancement (TFCE) multiple comparison-corrected.

We had also compared the global gray matter volume between groups, and there was no significant difference in the total brain volume between the HIV+ group and the HIV− group (including the total gray matter volume, total white matter volume, and the sum of gray and white matter volumes) (the effects of age and gender were removed).

### Within-group global network measures

**Figure 2:**
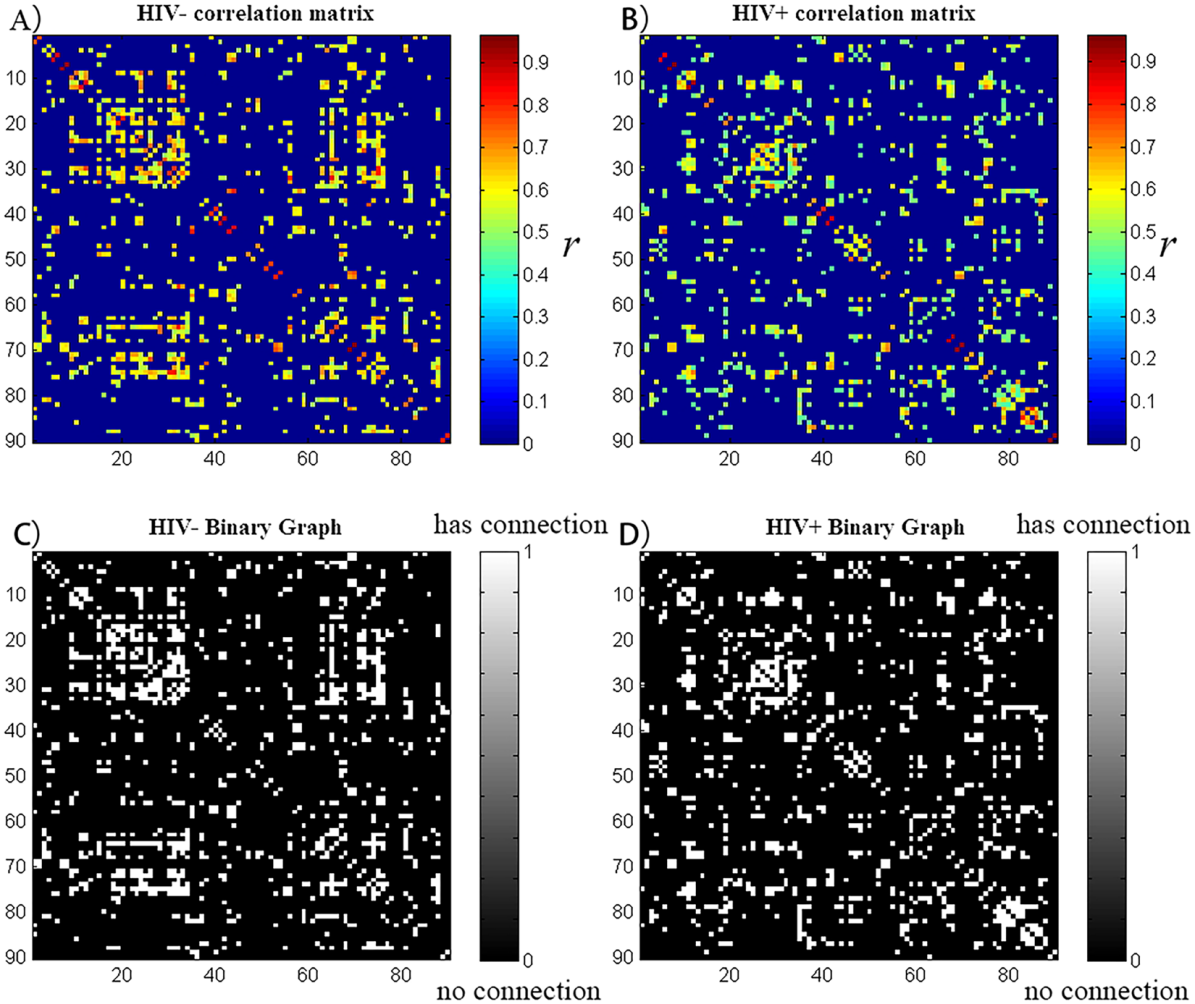
Correlation matrix and binary graph. HIV+ and HIV− groups’ correlation matrix; the strength of the connections is indicated by the color-bar. HIV+ and HIV− groups’ binary graph; the presence of a connection is indicated by the white-gray color. These matrices are graphs of the D_min_ (13%) threshold, where all nodes are fully connected in the two groups’ structural networks.

The minimum network density of the network fragmentation was D_min_ = 0.13. In order to study the changes of the network topology as a function of network density, we thresholded the constructed association matrices at a range of 0.13–0.45. The changes in global network measurements as a function of the network cost are shown in Fig. 2. The two networks followed a small-world organization with a wide range of network densities; both networks had a slightly higher path length than random networks with a substantially increased clustering coefficient compared to random networks. This pattern resulted in a small-world index greater than 1 across the range of network densities.

### Between-group differences in global network measures

#### Differences across network densities

**Figure 3:**
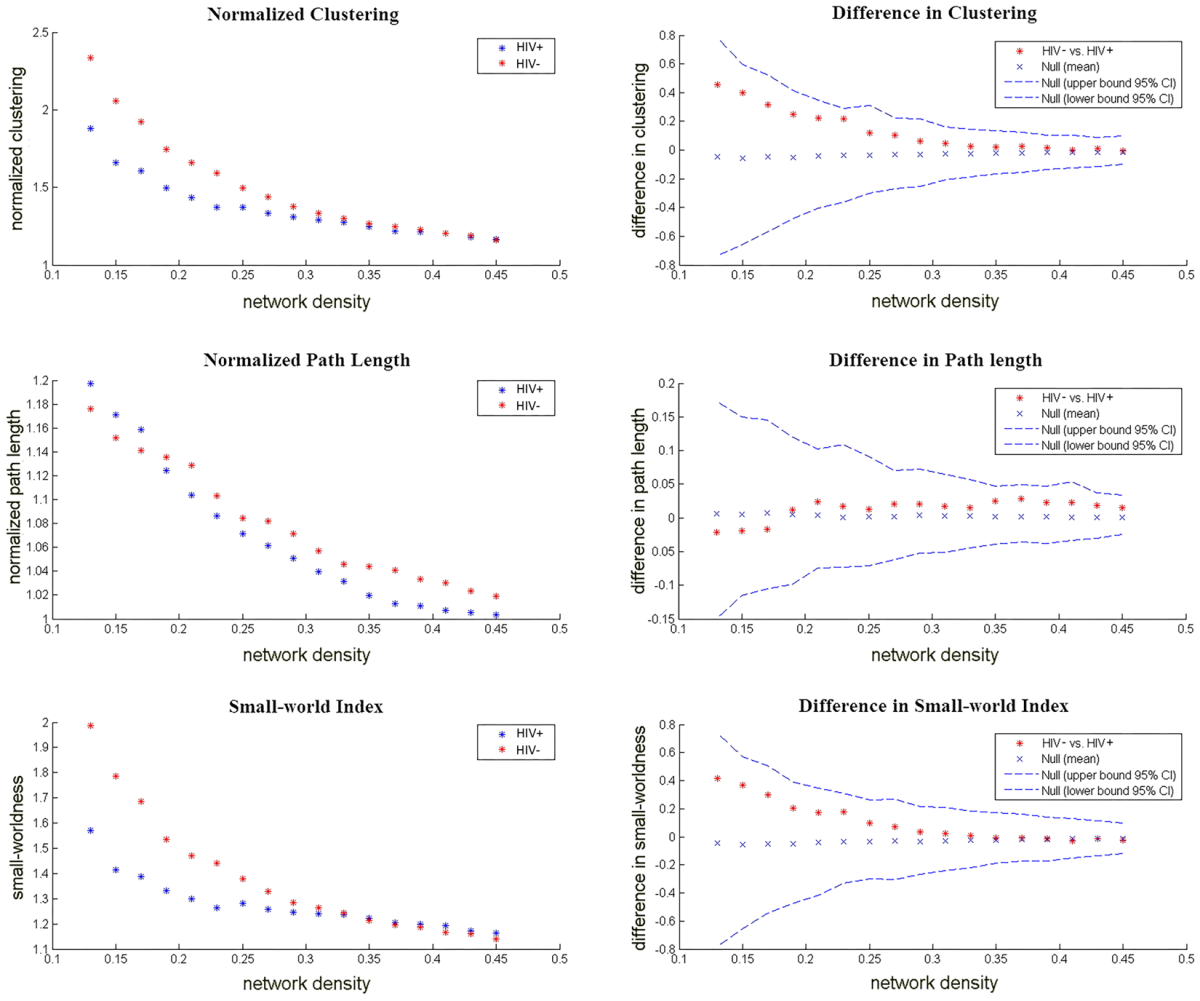
Changes in global network measurements and intergroup differences as a function of network density. HIV+ and HIV− networks’ normalized clustering (left top), normalized path length (left middle), and small-world index (left bottom). The intergroup differences and 95% confidence intervals in the normalized clustering (right top), normalized path length (right middle) and small-world index (right bottom). The difference is indicated by the red marker between the HIV− and HIV+ networks.

We investigated the differences between groups in the global network of networks at a range of densities (0.13:0.01:0.45) threshold (Fig. 3). The two networks exhibit a small-world organization, that is, a normalized clustering greater than one and a normalized path length close to one. Positive values indicate HIV− > HIV+, and negative values indicate HIV− < HIV+. Compared with HIV−, the HIV+ network was not significantly different regarding the normalized clustering, normalized path length or small-world index across a range of densities (p > 0.05).

### AUC analysis of global network measures

In addition to the comparison of networks at different densities, we compared the AUC (density range, 0.13:0.01:0.45) of the global network measurement curves between groups. Similar to identified differences across densities, HIV+ network did not exhibit a significantly different AUC for normalized clustering or small-worldness compared with the HIV− network.

### Between-group differences in regional network measures

**Figure 4:**
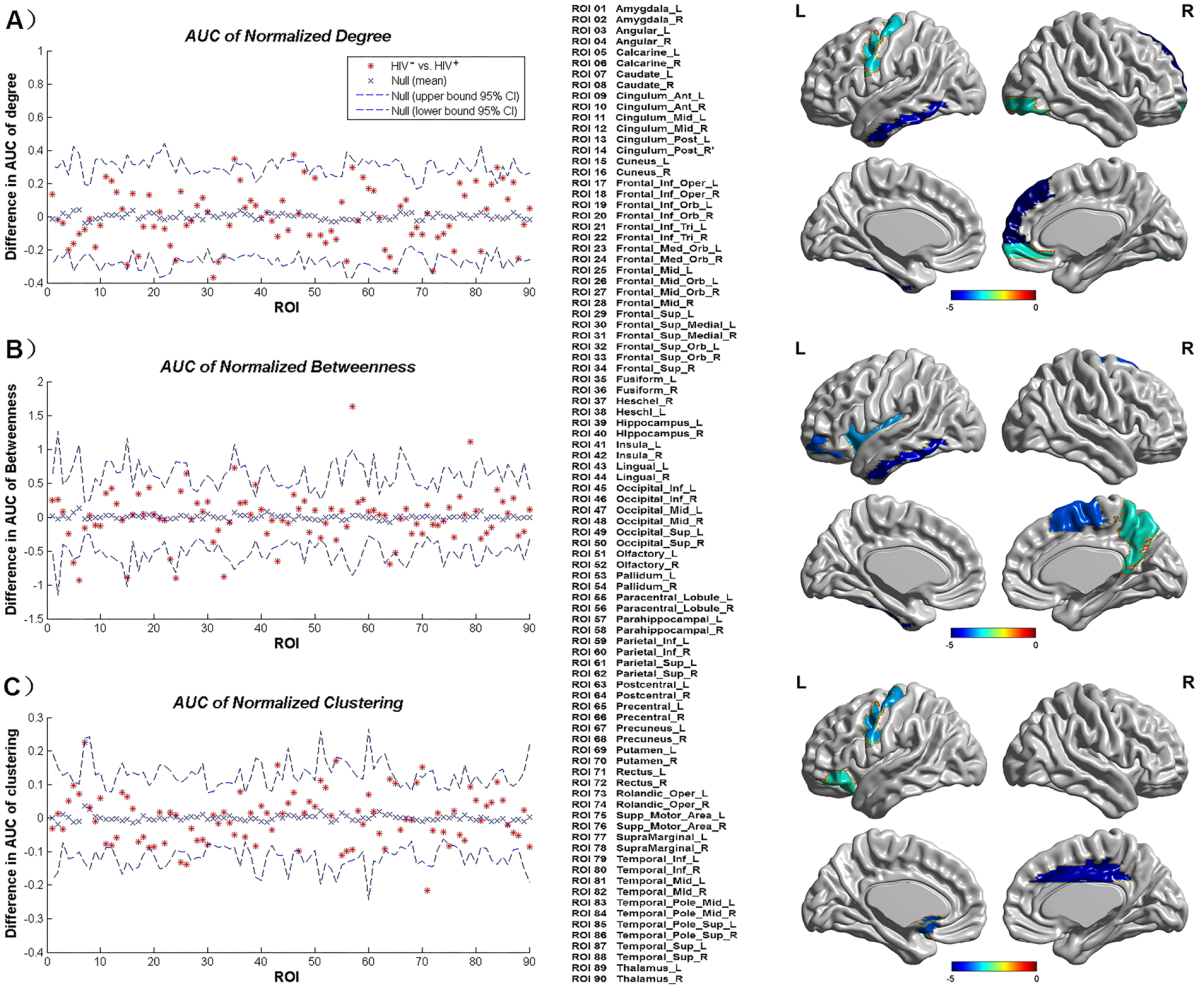
(**A**) Regions about the p_auc_MDeg compared the HIV+ group to the HIV− group. (**B**) Regions about the p_auc_MNodeBetw compared the HIV+ group to the HIV− group. (**C**) Regions about the p_auc_MClust compared the HIV+ group to the HIV− group. All regions survived following FDR correction (p < 0.05).

An AUC analysis was performed on the regional network measures. We also compared the AUC (density range, 0.13:0.01:0.45) of the global network measurement curves between groups (Fig. 4). Regions about the p_auc_MDeg, including the left fusiform gyrus, right inferior occipital gyrus, left inferior temporal gyrus, and right middle temporal gyrus, were significantly smaller in the HIV+ group, and the right medial orbital and right superior frontal gyrus exhibited a significantly greater degree in the HIV+ group (Fig. 4A). Regions about the p_auc_MNodeBetw, including the left hippocampus, left parahippocampal gyrus, and left inferior temporal gyrus, exhibited significantly smaller betweenness in the HIV+ group, and the bilateral calcarine fissure, right medial orbital, right superior frontal gyrus, left lingual gyrus, and right postcentral gyrus exhibited significantly greater betweenness in the HIV+ group (Fig. 4B). Regions about the p_auc_MClust, including the left lingual gyrus, right putamen, and left supplementary motor area, exhibited significantly smaller clustering in the HIV+ group, and left middle frontal gyrus, left superior frontal gyrus, left middle occipital gyrus, left gyrus rectus exhibited significantly greater clustering in the HIV+ group (Fig. 4C). All regions survived following FDR correction (p < 0.05).

### Network hubs

**Figure 5:**
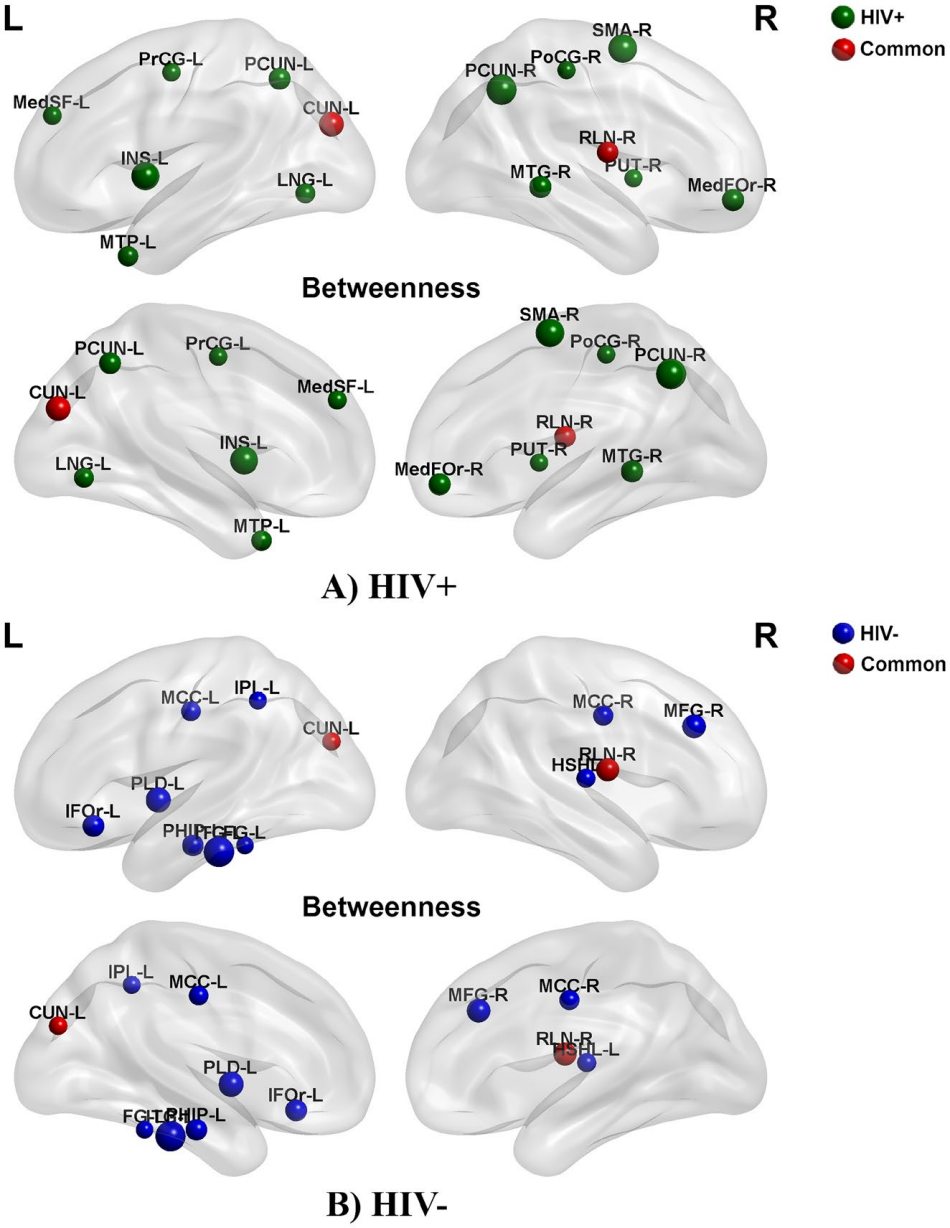
Network hubs. Green highlights hubs specific to HIV+ network; blue indicates hubs specific to HIV− network; red represents the common hubs in both groups.

If the regional betweenness centrality of a node was one SD higher than the average network betweenness, we considered it a hub. For the networks hubs were quantified at the threshold of D_min_ and based on the nodal betweenness curves’ AUC (density range, 0.13:0.01:0.45) (Fig. 5). For networks thresholded at D_min_, the common hubs in the both groups include the left cuneus cortex and right rolandic operculum; HIV+ network hubs of betweenness were identified in right middle frontal gyrus, right superior frontal gyrus, left insula, left lingual gyrus, right postcentral gyrus, left precentral gyrus, bilateral precuneus, right putamen, right supplementary motor gyrus and bilateral middle temporal gyrus, whereas the HIV− network hubs were identified in bilateral median cingulate, left inferior frontal gyrus, left middle frontal gyrus, left fusiform gyrus, right heschl gyrus, left pallidum, left parahippocampal gyrus, left inferior parietal and left inferior temporal gyrus.

### Random failure and targeted attack analysis

**Figure 6:**
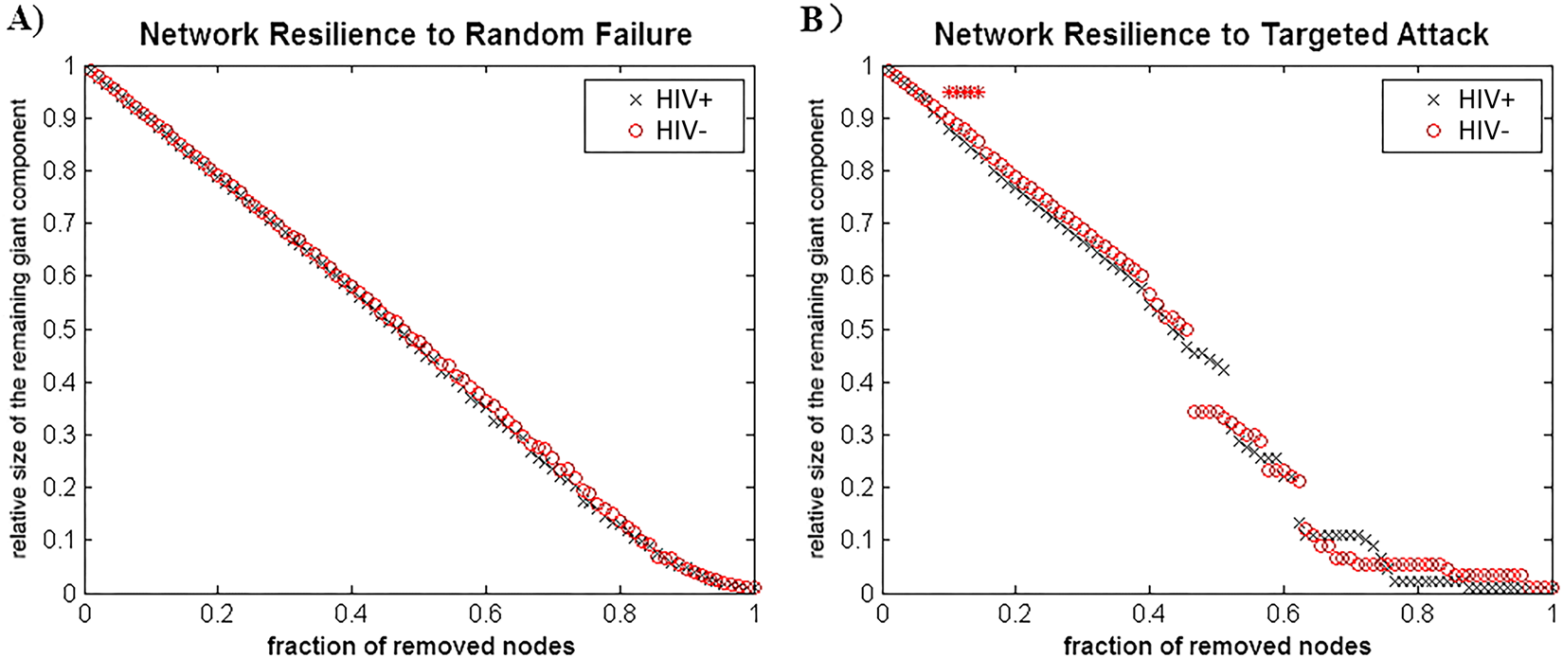
Random failure and targeted attack analysis. The changes in the size of the remaining maximum component of the network as a function of the fraction of nodes that are randomly removed are depicted (left). By removing the nodes in a rank order of decreasing nodal betweenness centrality, the same procedure is used to analyze the network response to targeted attack (right).

We calculated the size of the largest remaining component in response to the successive removal of nodes in random order in order to analyze the response of the networks to random failure. The results are shown in Fig. 6. In all parts of the removed nodes, the resilience of the random failure of the HIV+ network was not significant (p > 0.05) compared to HIV− (left). The AUC of the curve in the HIV+ network also increased compared to the HIV− network; however, this effect was not significant. In general, to targeted attack the HIV+ network was less robust than HIV− network, and the difference is significant (p < 0.05) at several fractions of attacked nodes.

### Correlation results

**Figure 7:**
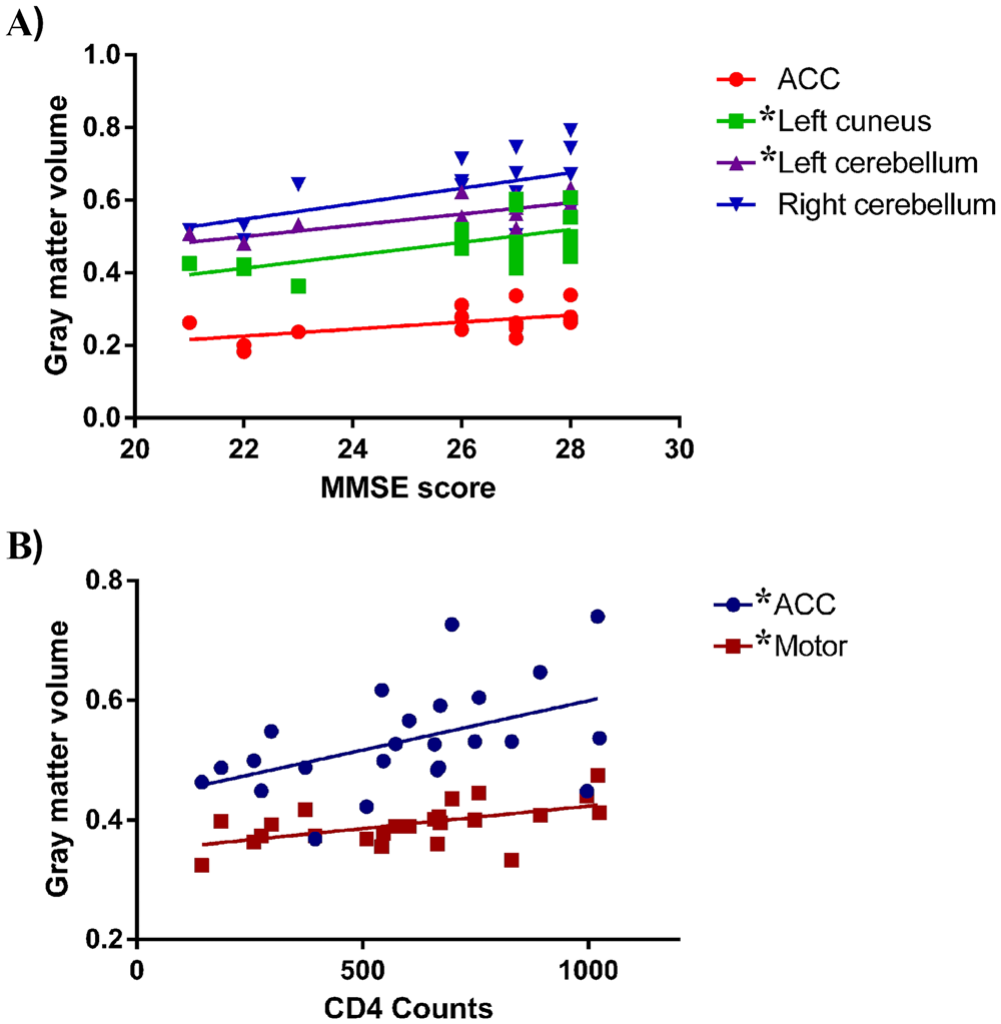
Correlation results (after Bonferroni-corrected for multiple comparisons). ACC: anterior cingulate cortex.

Using a linear regression analysis, we determined the volumes of specific gray matter regions were positively correlated with the MMSE scores and CD4 cell counts in the HIV-infected adolescents. The results are shown in Fig. 7. The MMSE scores were positively correlated with the GMV in the left cuneus and left cerebellum (ACC: r = 0.552, p = 0.018; left cuneus: r = 0.594, *p = 0.009; left cerebellum: r = 0.737, *p = 0.001; right cerebellum: r = 0.567, p = 0.014; Fig. 7A); the CD4 cell counts were also positively correlated with the GMV in the ACC and the sensorimotor area (ACC: r = 0.480, *p = 0.018; sensorimotor: r = 0.551, *p = 0.005; Fig. 7B).

## Discussion

We investigated the differences in the GMV covariance networks between HIV vertically transmitted adolescents and an HIV-exposed-uninfected group to confirm the effects of HIV on the developing brain. Specifically, we determined that HIV vertically transmitted adolescents exhibit: (1) GMV decreases across several cortical and subcortical regions that integrate cognitive, sensorimotor, and integrative roles; (2) disrupted node properties in the structural network and a shift in the hub distribution; and (3) significant correlations between the decreased GMV and the MMSE scores and CD4 cell counts. These findings indicate that abnormal gray matter integrity and nodal properties of structural wirings are associated with HIV infection in the developing brain.

Consistent with a previous volumetric MRI study^12^, a main finding of our VBM comparison was the decreased GMV in the ACC in the adolescents with vertically transmitted HIV. But the findings did not agree with an earlier paper^10^ where GMV in several areas were enlarged. It may not be a difference in methods of analysis but rather that so many of patients had HIV encephalopathy or school difficulties in the study by Sarma *et al*.^13^. In further correlation analyses, lower current CD4 cell counts were associated with gray matter atrophy within the ACC. It is believed that cingulate is part of the limbic system and injury in this brain site may yield the characteristic state of deficits in memory storage. The involvement of the ACC has been highlighted in relation to HIV-related pathology. Evidence from neuroimaging studies^24^ have linked HIV infection and ACC changes that may affect structure, cerebral flow, diffusion, and metabolism. For example, a PET study^25^ demonstrated reduced glucose uptake in the ACC and the mesial frontal gyrus in HIV+ individuals with undetectable plasma viral loads. In a task fMRI study^26^, alterations in ACC activation also partially accounted for verbal and memory deficits in women with HIV who had also used cocaine. Overall, these findings suggest that altered ACC gray matter integrity may represent a promising biomarker of cognitive dysfunction following HIV infection.

In the global network measures comparison, there was no significant difference in the small-worldness between the HIV-infected and HIV-exposed-uninfected subjects. This may be due to insignificant differences in the global subcortical volume between pediatric HIV patients and healthy controls. In addition, HIV-infected and HIV-exposed-uninfected adolescents share a similar living environment and may not develop distinct wiring patterns in anatomical networks that are sufficiently different from those of healthy controls to induce changes in structural covariance plasticity despite tremendous differences in HIV infection. Therefore, we could not elucidate the predispositions for or consequences of HIV infection regarding the large-scale brain anatomical network between the HIV-positive and HIV-negative subjects. Thus, it may be concluded that HIV+ adolescents and HIV− controls do not differ in global parameters of brain anatomical covariance. Both the HIV-infected and HIV-exposed-uninfected networks exhibited a small-world organization across a wide range of densities (Fig. 3) and AUC analyses. Previous studies have consistently demonstrated “small-world” architecture in structural brain networks during typical development in healthy subjects^27–29^. This network organization is thought to enable efficient information processing by providing an optimal balance between segregation and integration^30^. In the current study, we did not identify significant inter-group differences across the main small-worldness indexes, i.e., normalized clustering, normalized path length and small-worldness. Despite the lack of significant inter-group differences, these measures were lower than the reported literature^22^.

In regional network measures we identified between-group differences across several brain systems, which were consistent with previous findings. Several regions in the prefrontal cortex exhibited increased centrality (clustering, betweenness, and degree centrality) in the HIV+ network compared with the HIV-exposed-uninfected network. As an element of brain networks, individual nodes have unique centralities that are thought to be crucial for defining functional specialization^21,31^. Numerous neuropsychological studies have demonstrated subtle long-term neurocognitive deficits, specifically in cognitive functions subserved by the prefrontal and temporal cortices, in HIV-infected subjects^32^. Our recent work^33^ also identified reduced white matter integrity in the frontal and temporal regions in HIV-infected subjects. These studies may explain the enhanced centrality in the frontal regions and the reduced centrality in the temporal regions in the HIV+ network, which suggest disrupted anatomical interactions between the frontal and temporal regions and the rest of the brain.

The regions identified with hubs in HIV− versus HIV+ network appear to be involved in multi-scale sensory processing, memory, attention, and social cognition. In contrast, areas where HIV+ network centrality are increased seem to be involved in self-awareness and sensorimotor functions. For example, the anterior insula contains an interoceptive representation that provides the basis for all subjective sensations of the body^34,35^. The anterior insula is usually activated in conjunction with the ACC, and these two structures act as limbic sensory and motor cortices that producing salient sensations and motivations, respectively^34,36^. In addition, we determined that the shared hubs between the two groups were limited to the left cuneus cortex and the right rolandic operculum. We identified an increased hub number with a shift to non-hub regions identified in typical development in the HIV+ group. This finding may be correlated with the variable across individuals.

Substantial evidence suggests that global gray matter atrophy is associated with focal and global white matter damage, in part, due to axonal transection and subsequent retrograde neuronal loss^37,38^. HAART can effectively inhibit the burden of the HIV system; however, poor penetration into the CNS provides imperfect protection. In addition, HIV treatment is provided to patients with <350 CD4+ T cells/mm^3^ or plasma HIV ribonucleic acid levels of >55,000 copies/mL^39^, so early and long-term HIV treatment may be due to more virulent strains, resulting in direct viral effects on the GM by the very heterogeneous virus population. In our study, the average age at which antiretroviral therapy was initiated in our subjects was 8.5 years, and there was probably considerable brain damage. In general, we infer that the cerebral maturation process of HIV-infected adolescents might be affected by the direct viral effects to a greater extent. Thus, changes in the identified network-level in structural correlation network of HIV+ patients may be caused by neurotoxic effects on cortico-cortical connections. Previous diffusion tensor imaging studies reported a diffuse pattern of microstructure damage to white matter also supported the idea^33^.

For random failure and targeted attack analysis, the AUC of the random failure curve in the HIV infection network was not significantly different from that of the HIV-exposed-uninfected network, which suggests a similar resilience of both networks in response to random failure. However, the resilience of the HIV infection network was lower in response to targeted attack, and there was a significant difference between the groups in specific nodes. The more regularized networks are less resilient to random failure and exhibit reduced resilience to pathologies^40^. This observation is consistent with the nodal network measures results that suggest the HIV-exposed-uninfected network has less central hubs than the HIV-infected network because hubs in the structural connectome are thought to be energy-demanding and vulnerable to major diseases. A more recent study^41^ that compared cortical surface profiles and their structural connectivity changes between pediatric HIV patients and healthy controls also reported similar findings, but the main difference between this study and our research is the choice of appropriate healthy controls.

### Limitations

There are several limitations in the current study. First, because of the lack of healthy subjects with typical development, we could not draw conclusions regarding the role of HIV infection and antiviral treatment on the typical developing brain. Second, we lack more detailed clinical and cognitive measures to relate the alterations in gray matter integrity and structural covariance with functions in cognitive and motor domains. Third, we cannot distinguish our current results as a consequence of HIV infection or antiretroviral treatment.

## Conclusion

In summary, this investigation is the first study to use the graph analysis to investigate alterations in the GMV correlation networks between HIV vertically infected adolescents and matched HIV-exposed-uninfected subjects (the father, mother, or both parents were infected with HIV). We suggest a focal loss of gray matter, disrupted nodal profiles of structural wirings, and a shift in hub distribution may represent neuroanatomical biomarkers of HIV infection on the developing brain.
